# Clinical characteristics and predictors of esophagogastric variceal bleeding among patients with HCV-induced liver cirrhosis: An observational comparative study

**DOI:** 10.1371/journal.pone.0275373

**Published:** 2022-10-13

**Authors:** Saad El Deen Mohamed El Sheref, Shimaa Afify, Mahmoud S. Berengy

**Affiliations:** 1 Internal Medicine Department, Faculty of Medicine, Al-Azhar University Hospital, New Damietta, Egypt; 2 Gastroenterology Department, National Hepatology and Tropical Medicine Research Institute, Cairo, Egypt; Al-Azhar University, EGYPT

## Abstract

**Objectives:**

To investigate the clinical characteristics, risk factors, and predictors of esophagogastric variceal bleeding in patients with hepatitis C virus (HCV)-induced liver cirrhosis.

**Methods:**

This comparative observational study was carried out on 100 patients suffering from post hepatitis cirrhosis and portal hypertension who were admitted to the Internal Medicine Department, Al-Azhar University Hospital, Damietta Egypt. Patients were classified into two groups: 50 of them presented with esophagogastric varices with acute variceal bleeding, and 50 patients presented without bleeding. Data were collected, coded, revised, and entered into the Stata software version 16.

**Results:**

The mean age of patients with bleeding was slightly higher than those without bleeding (55.58 ± 5.89 vs. 52.54 ± 9.01 years), p = 0.049. Mild ascites, positive H.Pylori, and Child-Pugh score B and C were an independent predictors of esophagogastric variceal bleeding (OR = 0.036, 95% CI: 0.0004–0.36; p = 0.005), (OR = 7.36, 95% CI: 1.44–37.59; p = 0.016), (OR = 19.0, 95% CI: 2.02–186.3; p = 0.010), and (OR = 40.51, 95% CI: 2.18–751.31; p = 0.013). The sensitivity of this model was 93.88%, specificity was 53.85%, PPV was 88.46%, NPV was 70.0%, correctly classified patients were 85.48%, and AUC was 90.27%. In the second model, pepsinogen level higher than 43.5 μg/l, AST (>54.5), Bilirubin (>1.45), and Hemoglobin (>11.5) were a significant independent predictors of esophagogastric variceal bleeding (OR = 1.18, 95% CI: 1.09–1.27; p<0.001), (OR = 1.14, 95% CI: 1.03–1.27; p = 0.007), (OR = 5.55, 95% CI: 1.21–25.43; p = 0.027), and (OR = 0.05, 95% CI: 0.008–0.32; p = 0.002), respectively. The sensitivity of this model was 92%, specificity was 98%, PPV was 97.87%, NPV was 92.45%, correctly classified patients were 95%, and AUC was 98.68%.

**Conclusion:**

The independent predictors of esophagogastric variceal bleeding were ascites, positive H. pylori, Child-Pugh score B and C, pepsinogen level higher than 43.5 μg/l, AST (>54.5), bilirubin (>1.45), and hemoglobin (>11.5). Laboratory investigations are more reliable in predicting variceal bleeding and excluding non-variceal bleeding; however, clinical symptoms should not be neglected, especially H. pylori infection, ascites, and Child-Pugh score.

## Introduction

Portal hypertension is a clinical condition caused by a pathological elevation in portal vein pressure attributed to a variety of factors, the most prevalent of which are liver cirrhosis in the Western world and schistosomiasis in the African continent [1, 2]. In patients with liver cirrhosis, bleeding from esophageal and gastric varices is a fatal consequence of portal hypertension [3]. Gastric variceal bleeding is more severe than esophageal variceal bleeding, but it is less common [4]. Esophageal varices are present in about 50% of patients with cirrhosis, whereas gastric varices, either alone or in combination with esophageal varices, are seen in roughly 20% of cirrhotic individuals [5, 6]. Variceal bleeding occurs in 16% to 75.6% of patients who have not previously undergone treatment for esophageal varices and in 25% of patients with gastric varices [7]. However, this prevalence may change according to the liver disease status (compensated or decompensated) and timing of treatment administration (early or late).

Budd-Chiari syndrome, constrictive pericarditis, severe right-sided heart failure massive splenomegaly, portal vein obstruction, alpha-1 antitrypsin deficiency, and primary biliary cirrhosis are among the causes of portal hypertension, which may lead to esophageal varices [8]. Varices are most superficial and have the weakest wall near the gastroesophageal junction; therefore, bleeding is more common there. Approximately half of all acute variceal bleeding ceases spontaneously [9]. High hepatic venous pressure gradient, wide varices, alcohol-related cirrhosis, encephalopathy, thrombocytopenia, and stomach variceal hemorrhage, are all risk factors for esophagogastric variceal bleeding [10–13]. Moreover, bleeding is more likely to occur in those over 60 years old, renal failure, excessive alcohol intake, wide esophageal varices, severe liver disease, and the development of a hepatoma [10, 14]. Infection with Helicobacter pylori (H. pylori) is a common cause of esophagogastric variceal bleeding [15, 16]. There are many studies on the association between H. pylori infection and hepatic encephalopathy, thrombocytopenia, portal hypertensive gastropathy, chronic atrophic gastritis, and peptic ulcer in patients with liver cirrhosis and portal hypertension have been published [15–19]. In this study, we aimed to investigate the clinical characteristics, risk factors, and predictors of esophagogastric variceal bleeding in patients with HCV-induced liver cirrhosis.

## Methods

### Study design

This comparative observational study was carried out from September 2018 to April 2020 on 100 patients suffering from post-hepatitis cirrhosis and portal hypertension who visited the Internal Medicine Department, Al-Azhar University Hospital, Damietta, Egypt. Patients were classified into two groups: 50 of them presented with esophagogastric varices with acute variceal bleeding, and 50 patients presented without bleeding. The study protocol was approved by the ethical committee of the Al-Azhar University hospital (IRB:00012367-18-07-007). All patients provided written and verbal informed consent before participating in this study, and their personal data were fully anonymized.

### Inclusion and exclusion criteria

Patients were included if they had HCV-induced cirrhosis based on clinical criteria, including history, physical examination, laboratory parameters, and imaging findings. We excluded the patients who were using drugs for portal hypertension, such as propranolol, and those who were using anticancer agents or non-steroidal anti-inflammatory drugs (NSAIDs). In addition, patients who had gastrectomy and terminally ill patients with liver cirrhosis and hepatocellular carcinoma were excluded.

### Data collection and patients’ examination

All patients were subjected to the following:

1. Full medical history and thorough clinical examination with special stress on smoking, chronic illness, inflammation, assess body weight, blood pressure, history of anti-hepatitis c virus treatment, and examination of the liver (cirrhosis, splenomegaly, and signs of portal hypertension).
2. Laboratory investigations, including complete blood picture (CBC), liver function tests [serum bilirubin, serum albumin, serum alanine transferase (ALT) and aspartate transferase (AST)], International Normalized Ratio (INR), serum creatinine, and serum pepsinogen that was measured as indices of gastric acid secretion. Blood samples were taken after 12 to 14 hours of overnight fasting and centrifuged within 30 to 45 min of collection. Blood sample analysis was done at the Clinical Pathology Department and Research Laboratory at Al-Azhar University Hospital on the day of blood collection. Creatinine and uric acid were analyzed by a fully automated chemistry analyzer.
3. Pelvi-abdominal ultrasonography examinations were held in the Internal Medicine Sonography Unit to evaluate findings that suggest cirrhosis, measure the portal vein diameter, and measure the longitudinal (bipolar) diameter of the spleen. The diagnosis of cirrhosis is based on the combination of ultrasound abdomen and liver function tests (prothrombin time and serum albumin). The assessment of staging of the liver condition by the Child-Pugh classification.
4. Upper gastrointestinal endoscopy: All patients were subjected to an upper gastrointestinal endoscopy: all endoscopies were performed in the Internal Medicine Endoscopy unit by a staff member of the Internal Medicine Department of Al-Azhar University Hospital at new Damietta. Esophageal varices were graded according to their size; a grading classification of I–IV will be used:

**Grade I**: was used for varices in the level of the mucosa.

**Grade II**: for varices smaller than 5 mm filling less than 1/3 of the oesophageal lumen.

**Grade III**: for varices larger than 5mm filling more than 1/3 of the oesophageal lumen.

**Grade IV**: for varices occupied more than 2/3 of oesophageal lumen

The patients presented with acute variceal bleeding were subjected to management according to the management protocol for variceal bleeding, while those without bleeding will be subjected to management according to their condition. Endoscopic variceal ligation (EVL) or endoscopic injection sclerotherapy (EIS) with 5% ethanolamine oleate will be performed for bleeding from esophageal varices. Acute bleeding from gastric fundal varices was treated by endoscopic Histoacryl (n-butyl-2-cyanoacrylate [CA]; B. Braun, Melsungen, Germany) glue injection.

### Statistical analysis

Data were collected, coded, revised, and entered into the Stata software version 16. Qualitative data were presented as numbers and percentages, while the qualitative data were presented as mean and standard deviation (SD). Chi-square was used to compare two groups with qualitative data. An independent t-test was used to compare two groups in parametric quantitative data, and the Mann-Whitney test was in non-parametric distribution. Independent predictors of esophageal varices bleeding were detected using the univariate and multivariate logistic regression, and data were presented as odds ratio (OR) and 95% confidence interval (CI). A receiver operating characteristic (ROC) analysis was performed to estimate the sensitivity, specificity, positive predictive value (PPV), negative predictive value (NPV), and area under the curve (AUC). A p-value less than 0.05 was considered significant.

### Results

#### Demographic and clinical characteristics

The mean age of patients with bleeding was slightly higher than those without bleeding (55.58 ± 5.89 vs. 52.54 ± 9.01 years), p = 0.049. Both males and females were represented equally in both groups (p = 0.548). Among the patients with bleeding, 40% had severe ascites, 32% moderate ascites, and 28% mild ascites, while in the non-bleeding group, 20% had mild ascites, 4% moderate ascites, and 2% severe ascites (p<0.001). Lower limb edema was detected in 88% in the bleeding group and 26% in the non-bleeding group (p<0.001). The rate of jaundice was significantly higher in the bleeding group compared to the non-bleeding (18% vs. 4%, p = 0.025). On the other hand, the palpable liver was less common in the bleeding group compared to the non-bleeding group (10% vs. 76%, p<0.001). Regarding the Child-Pugh score, 2%, 40%, and 52% had scores A, B, and C among the bleeding group, while in the non-bleeding group, it was 64%, 28%, and 8%, respectively. Positive H.pylori was observed in 92% of the bleeding group, compared to 24% in the non-bleeding group (p<0.001). **Table 1** summarizes the clinical characteristics of included patients.

**Table 1:** Demographic and clinical characteristics.

Parameters		With Bleeding (n = 50)	Without Bleeding (n = 50)	P-value
Age, years		55.58 ± 5.89	52.54 ± 9.01	**0.049**
Gender	Male	27 (54.0%)	24 (48.0%)	0.548
	Female	23 (46.0%)	26 (52.0%)	
Ascites	No	0 (0%)	37 (74%)	**<0.001**
	Mild	14 (28%)	10 (20%)	
	Moderate	16 (32%)	2 (4%)	
	Severe	20 (40%)	1 (2%)	
Lower limb edema	No	6 (12%)	37 (74%)	**<0.001**
	Yes	44 (88%)	13 (26%)	
Jaundice	No	41 (82%)	48 (96%)	**0.025**
	Yes	9 (18%)	2 (4%)	
Others Liver cell failure	Pruritus	13 (26%)	2 (4%)	0.284
	Palmer erythema	17 (34%)	8 (16%)	
	Caput medusa	20 (40%)	4 (8%)	
Liver	Non palpable	45 (90%)	12 (24%)	**<0.001**
	Palpable	5 (10%)	38 (76%)	
Splenomegaly	No	0 (0%)	0 (0%)	1.00
	Yes	50 (100%)	50 (100%)	
H. pylori	Negative	4 (8%)	38 (76%)	**<0.001**
	Positive	46 (92%)	12 (24%)	
Liver cirrhosis	Mild	0 (0%)	38 (76%)	**<0.001**
	Mild to moderate	5 (10%)	8 (16%)	
	Moderate	19 (38%)	0 (0%)	
	Severe	26 (52%)	4 (8%)	
Child classification	A	1 (2%)	32 (64%)	**<0.001**
	B	20 (40%)	14 (28%)	
	C	29 (58%)	4 (8%)	
Encephalopathy	0	15 (30%)	42 (84%)	**<0.001**
	I	9 (18%)	5 (10%)	
	II	15 (30%)	2 (4%)	
	III	11 (22%)	1 (2%)	
HCV treatment	Not received	9 (18%)	0 (0%)	**<0.001**
	Prepared	0 (0%)	50 (100%)	
	Received	41 (82%)	0 (0%)	

HCV: Hepatitis C virus, H.pylori: Helicobacter pylori

### Laboratory findings

In terms of CBC, patients with bleeding were presented with significantly lower hemoglobin (p<0.001), RBCs (p = 0.010), and platelet (p<0.001) compared with the non-bleeding group. On the other hand, the serum levels of ALT, AST, and bilirubin were significantly (p<0.001) higher in the bleeding group; however, the concentration of albumin was significantly lower (p<0.001). Regarding serum creatinine, it was substantially higher (p = 0.004) in the bleeding group than in the non-bleeding group. Moreover, patients with bleeding were associated with a higher level of Pepsinogen and INR compared to those without bleeding (p<0.001). **Table 2** shows the laboratory findings of both groups.

**Table 2:** Laboratory findings.

Parameters			With Bleeding (n = 50)	Without Bleeding (n = 50)	P-value
CBC	Hemoglobin, g/dl		9.01±2.22	11.87±2.17	**<0.001**
	WBCs × 109/L		6.46±2.57	6.34±1.83	0.803
	RBCs × 1012/L		3.72±0.65	4.09±0.76	**0.010**
	Platelet × 109/L		96.38±41.45	162.34±71.46	**<0.001**
Liver function tests	AST, U/L		87.80 ±39.13	53.86±20.36	**<0.001**
	ALT, U/L		70.56 ±33.86	45.30±17.57	**<0.001**
	Serum bilirubin, mg/dl		2.08±0.73	1.30±0.58	**<0.001**
	Serum albumin, mg/dl		3.09±0.39	3.56±0.55	**<0.001**
Renal function tests	Serum creatinine, mg/dl		1.30±0.43	1.09±0.27	**0.004**
Pepsinogen			67.30±13.84	38.58±10.31	**<0.001**
INR			1.88±0.41	1.45±0.38	**<0.001**
Blood pressure		SBP	100.8±17.68	120.8±7.51	**<0.001**
		DBP	66.2±11.84	77.9±5.81	**<0.001**

INR: international normalized ratio, SBP: systolic blood pressure, DBP: Diastolic blood pressure, ALT: Alanine transaminase, AST: Aspartate transaminase, WBC: White blood cell, RBC: Red blood cell

### Characteristics of esophageal varices

In the bleeding group, the most common grade of Esophageal Varices was grade III (70%), followed by grade II (24%) and IV (6%), while in the non-bleeding group, the most common grade was 0 (80%), followed by grade I (8%), III (6%), II (4%), and IV (2%), with a significant difference between both groups (p<0.001). Injection Sclerotherapy and band ligation occurred in 76% and 24% in the bleeding group, respectively. In the non-bleeding group, follow-up was the most common followed approach (88%), followed by band ligation (6%) and injection sclerotherapy (6%). Dilated portal hypertension was positive in all patients in both groups (**Table 3**).

**Table 3:** Esophageal varices profile.

Parameters		With Bleeding (n = 50)	Without Bleeding (n = 50)	P-value
Esophageal Varices grading	0	0 (0%)	40 (80%)	**<0.001**
	I	0 (0%)	4 (8%)	
	II	12 (24%)	2 (4%)	
	III	35 (70%)	3 (6%)	
	IV	3 (6%)	1 (2%)	
Intervention made	Band ligation	12 (24%)	3 (6%)	**<0.001**
	Follow up	0 (0%)	44 (88%)	
	Injection Sclerotherapy	38 (76.0%)	3 (6%)	
Portal HTN	Negative	0 (0%)	0 (0%)	1.00
	Positive	50 (100%)	50 (100%)	

### Predictors of esophageal varices bleeding

Univariate logistic regression showed a significant association between esophagogastric variceal bleeding and mild ascites (OR = 0.07, 95% CI: 0.008–0.610; p = 0.016), LL edema (OR = 20.87, 95% CI: 7.22–60.33; p<0.001), Jaundice (OR = 5.26, 95% CI: 1.07–25.77; p = 0.040), palpable liver (OR = 0.03, 95% CI: 0.011–0.108; p<0.001), and mild to moderate liver cirrhosis (OR = 0.096, 95% CI: 0.020–0.446; p = 0.003). Similarly, patients with positive H.pylori were associated with significantly higher risk of bleeding (OR = 36.41, 95% CI: 10.85–122.17; p<0.001). Patients with elevated levels of pepsinogen, bilirubin, creatinine, AST, ALT, and INR were associated with increased risk of bleeding (OR = 1.15, 95% CI: 1.09–1.22; p<0.001), (OR = 10.60, 95% CI: 3.62–30.99; p<0.001), (OR = 5.79, 95% CI: 1.61–20.8; p = 0.007), (OR = 1.04, 95% CI: 1.02–1.07; p<0.001), (OR = 1.04, 95% CI: 1.02–1.07; p<0.001), and (OR = 14.11, 95% CI: 4.29–46.42; p<0.001), respectively (**Table 4**).

**Table 4:** Univariate logistic regression.

Variable		OR (95% CI)	P-value
Age	Old	1.05 (0.99–1.11)	0.052
Gender	Male	1.27 (0.57–2.79)	0.549
Ascites	Mild	0.07 (0.008–0.610)	**0.016**
	Moderate	0.50 (0.033–4.818)	0.471
LL edema	Positive	20.87 (7.22–60.33)	**<0.001**
Jaundice	Positive	5.26 (1.07–25.77)	**0.040**
Liver	Palpable	0.03 (0.011–0.108)	**<0.001**
H.pylori	Positive	36.41 (10.85–122.17)	**<0.001**
Liver cirrhosis	Mild to moderate	0.096 (0.020–0.446)	**0.003**
Child Score	B	45.71 (5.57–374.90)	**<0.001**
	C	231.9 (24.49–2197.6)	**<0.001**
Encephalopathy	I	5.04 (1.45–17.45)	**0.011**
	II	22.4 (4.59–109.16)	**<0.001**
	III	28 (3.29–237.62)	**0.002**
SBP		0.89 (0.85–0.94)	**<0.001**
DBP		0.86 (0.82–0.92)	**<0.001**
Pepsinogen		1.15 (1.09–1.22)	**<0.001**
WBCs		1.02 (0.85–1.22)	0.801
RBCs		0.47 (0.26–0.85)	**0.013**
Platelet		0.97 (0.96–0.99)	**<0.001**
Bilirubin		10.60 (3.62–30.99)	**<0.001**
Serum Creatinine		5.79 (1.61–20.8)	**0.007**
Albumin		0.10 (0.033–0.314)	**<0.001**
AST		1.04 (1.02–1.07)	**<0.001**
ALT		1.04 (1.02–1.07)	**<0.001**
INR		14.11 (4.29–46.42)	**<0.001**
HG		0.35 (0.23–0.52)	**<0.001**
Esophageal Varices	II	2.0 (0.13–30.16)	0.617
	III	3.89 (0.30–49.90)	0.297
Treatment	Band ligation	0.31 (0.056–1.77)	0.191

INR: international normalized ratio, SBP: systolic blood pressure, DBP: Diastolic blood pressure, ALT: Alanine transaminase, AST: Aspartate transaminase, WBC: White blood cell, RBC: Red blood cell, HG: hemoglobin

**Fig 1:**
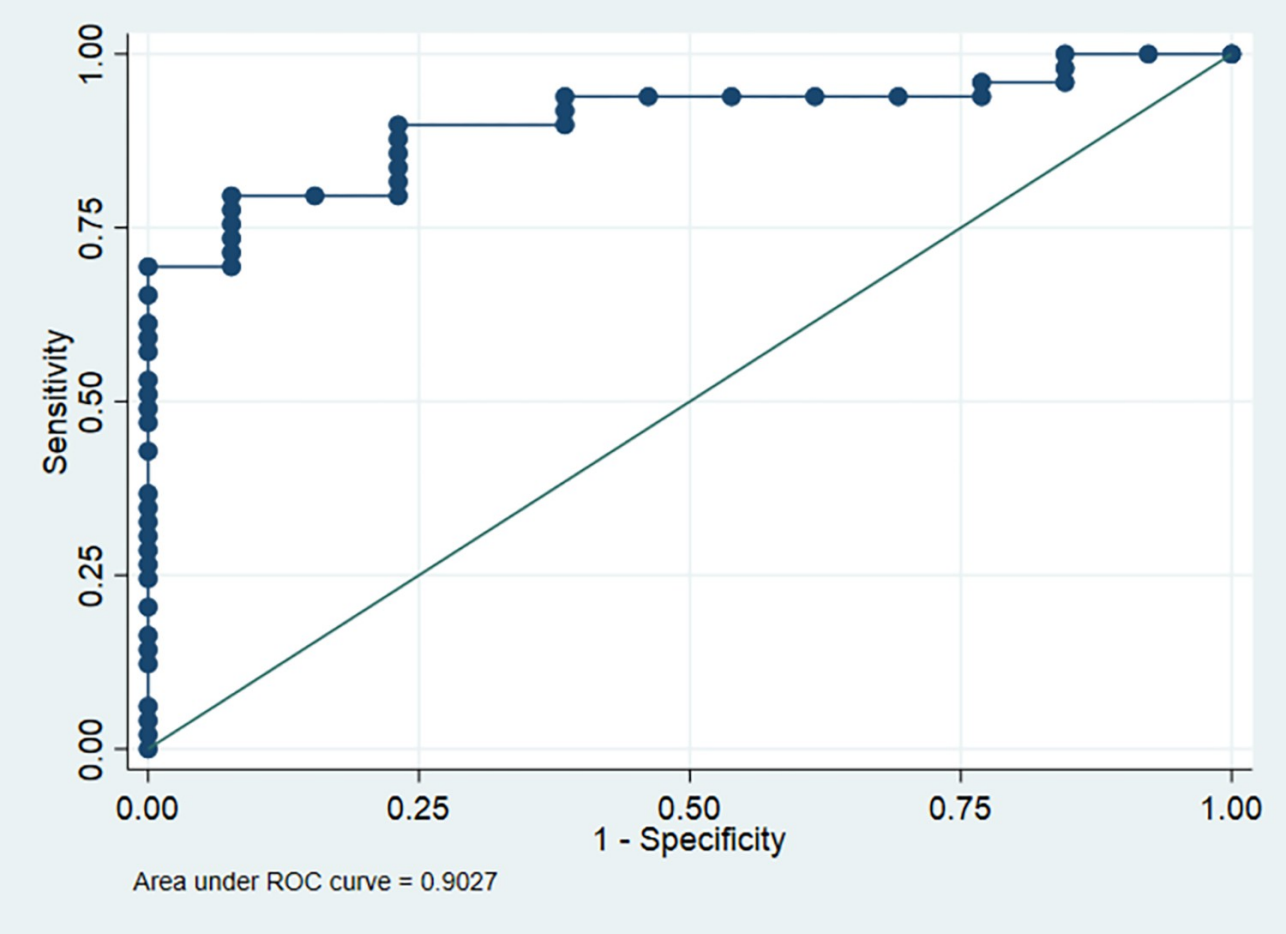
ROC curve of the first model (clinical symptoms).

We conducted a multivariate analysis based on two models; the first model included the significant clinical variables in univariate regression, and the second model included the significant laboratory variables in univariate regression. Our findings showed that mild ascites, positive H.Pylori, and Child-Pugh score B and C were an independent predictors of esophagogastric variceal bleeding (OR = 0.036, 95% CI: 0.0004–0.36; p = 0.005), (OR = 7.36, 95% CI: 1.44–37.59; p = 0.016), (OR = 19.0, 95% CI: 2.02–186.3; p = 0.010), and (OR = 40.51, 95% CI: 2.18–751.31; p = 0.013), **Table 5**. The sensitivity of this model was 93.88%, specificity was 53.85%, PPV was 88.46%, NPV was 70.0%, correctly classified patients were 85.48%, and AUC was 90.27% (**Fig 1**).

**Table 5:** Multivariate logistic regression.

Model	Variables		OR (95% CI)	P-value
Model 1	Ascites	Mild	0.036 (0.004–0.36)	**0.005**
	Jaundice	Positive	0.66 (0.08–4.93)	0.687
	Liver	Palpable	4.77 (0.45–50)	0.192
	H.pylori	Positive	7.36 (1.44–37.59)	**0.016**
	Child Score	B	19 (2.02–186.3)	**0.010**
		C	40.51 (2.18–751.31)	**0.013**
	Encephalopathy	I	0.46 (0.08–2.60)	0.382
		II	1.33 (0.13–13.22)	0.802
		III	1.43 (0.07–27.50)	0.810
	SBP (>122.5)		0.57 (0.26–1.26)	0.171
	DBP (>77.5)		1.28 (0.71–2.29)	0.404
Model 2	Platelet (>131.5)		1.00 (0.94–1.06)	0.964
	Pepsinogen (>43.5)		1.18 (1.09–1.27)	**<0.001**
	Serum Creatinine (>1.07)		1.74 (0.04–63.24)	0.756
	Albumin (>3.85)		0.41 (0.02–6.17)	0.520
	AST (>54.5)		1.14 (1.03–1.27)	**0.007**
	ALT (>49.5)		0.90 (0.77–1.05)	0.203
	INR (>1.55)		0.09 (0.006–1.44)	0.090
	Bilirubin (>1.45)		5.55 (1.21–25.43)	**0.027**
	Hemoglobin (>11.5)		0.05 (0.008–0.32)	**0.002**

Model 1 involves the clinical symptoms and scores, while model 2 shows the laboratory findings

**Fig 2:**
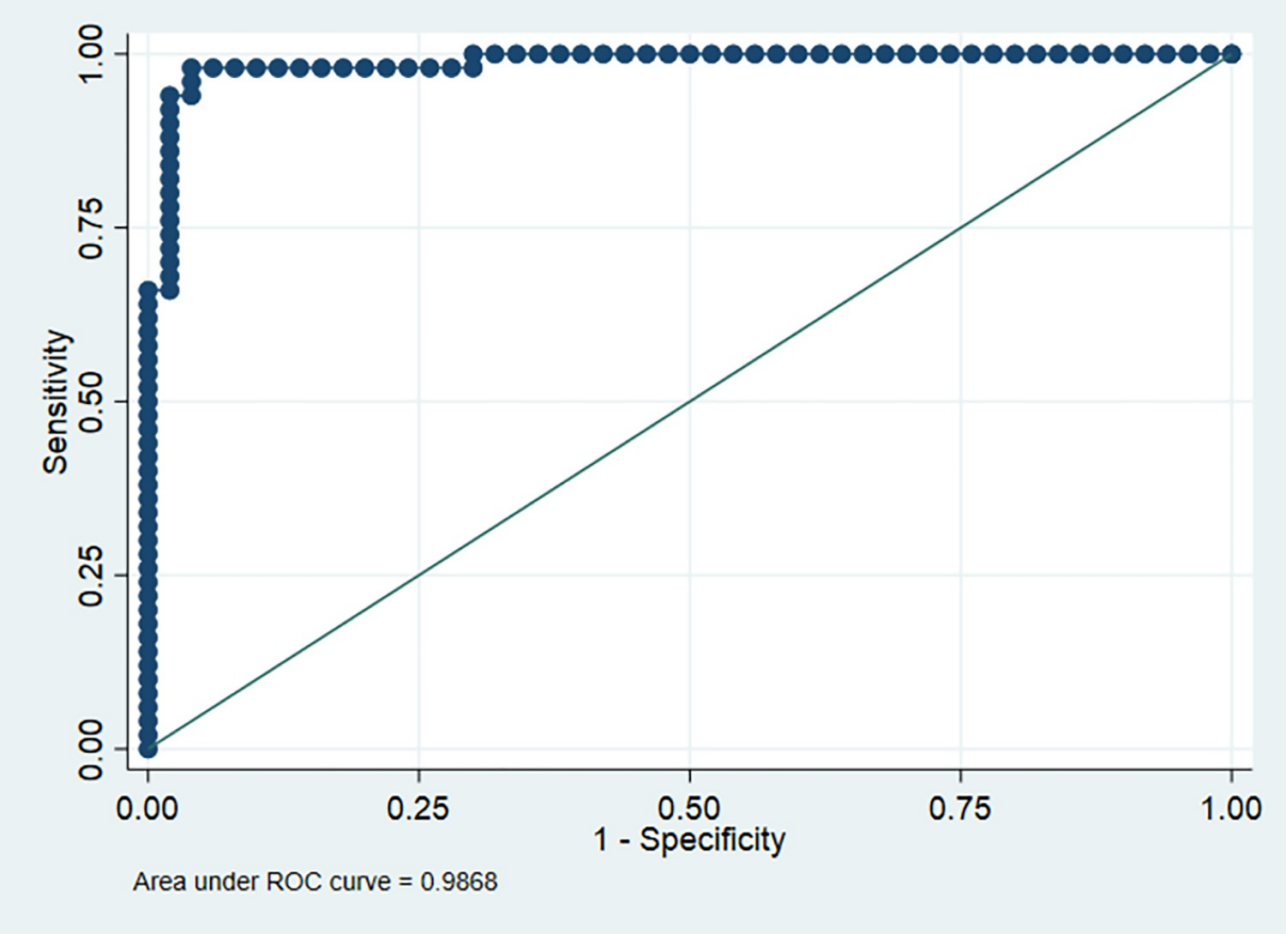
ROC curve of model 2 (laboratory profile).

In the second model, pepsinogen level higher than 43.5 μg/l, AST (>54.5), Bilirubin (>1.45), and Hemoglobin (>11.5) were a significant independent predictors of esophagogastric variceal bleeding (OR = 1.18, 95% CI: 1.09–1.27; p<0.001), (OR = 1.14, 95% CI: 1.03–1.27; p = 0.007), (OR = 5.55, 95% CI: 1.21–25.43; p = 0.027), and (OR = 0.05, 95% CI: 0.008–0.32; p = 0.002), respectively. The sensitivity of this model was 92%, specificity was 98%, PPV was 97.87%, NPV was 92.45%, correctly classified patients were 95%, and AUC was 98.68% (**Fig 2**).

## Discussion

In this study, our findings showed that patients with esophagogastric variceal bleeding were presented with moderate to severe ascites, lower limb edema, non-palpable liver, splenomegaly, dilated portal vein, advanced Child-Pugh score (B and C), and positive H. pylori. Moreover, their laboratory profile showed reduced hemoglobin, RBCs, platelets, albumin, and elevated ALT, AST, bilirubin, creatinine, INR, and pepsinogen.

Regarding the predictors of esophagogastric variceal bleeding, mild ascites, positive H. pylori, and Child-Pugh score B and C were independent predictors. In addition, pepsinogen levels higher than 43.5 μg/l, AST (>54.5), Bilirubin (>1.45), and Hemoglobin (>11.5) were significant independent predictors of esophagogastric variceal bleeding. The sensitivity, specificity, and accuracy of the second model (Laboratory) were significantly higher than the first model (Clinical), suggesting that laboratory investigations are more reliable in the prediction of variceal bleeding and excluding non-variceal bleeding; however, clinical symptoms should not be neglected, especially, H. pylori infection, ascites, and Child-Pugh score.

Similar to our findings, the Child-Pugh score, Model for End-Stage Liver Disease (MELD) score, ascites, and encephalopathy, were substantially (p<0.0001) different between patients with esophageal variceal bleeding due to liver cirrhosis who survived and those who died, according to Benedeto-Stojanov and colleagues. In the patients who died, bilirubin, serum creatinine, and INR were considerably greater, whereas albumin was significantly lower (p<0.0001) [20]. On the other hand, Tafarel et al. reported that there were no significant associations between esophageal variceal bleeding and Child-Pugh and MELD scores. However, they found that previous upper gastrointestinal bleeding and thrombocytopenia were significant independent predictors of esophageal variceal bleeding [21]. Garcia and Bosch investigated the risk factors for esophagogastric variceal rupture bleeding and concluded that stress, endotoxemia, hepatocellular carcinoma (HCC), ascites, and hepatic functional reserve are all significant factors [22]. Another study by Elsebaey et al. showed a significant difference between patients with gastric variceal bleeding and those without bleeding in terms of Child-Pugh score (p = 0.001). About 68% of the bleeding group had class C, 22.73% had class B, and 9.09% had class A, while in the non-bleeding group, 25% had class C, 53.13% class B, and 21.88% class A. In addition, they observed that 72.73% of the bleeding group had positive H. pylori, compared to 40.62% in the non-bleeding group (p = 0.0049). Moreover, patients in the bleeding group had a higher serum gastrin level than those without bleeding (p = 0.02). Finally, they concluded that Child-Pugh C score (OR = 5.325; p = 0.001), H. pylori (OR = 3.89; p = 0.005), and histological pattern of chronic gastritis (OR = 6.527; p = 0.037) are independent predictors of bleeding gastric varices [15]. In a French multicenter study, authors found that hemoglobin < 10 g/dL was significantly associated with increased risk of esophagogastric varices bleeding (OR: 1.7; 95%CI 1.1–3.3), which is similar to our findings [23].

Despite being developed more than 30 years ago, the Child-Pugh score is still regarded as the gold standard in cirrhotic patient prognosis. Nonetheless, it has certain limitations, such as clinical parameter subjectivity and low discriminatory power [24, 25]. Class A patients typically have a satisfactory median survival time, while Child-Pugh Class B patients are a diverse group, as their clinical status may be stable for more than a year or suddenly worsen [26, 27]. Patients in Child-Pugh Class C are the most common candidates for the surgery [28]. Previous studies have proposed a "combined score" using quantitative liver function tests to address the limitations of the Child-Pugh score or have used the scores that were initially created to measure multiorgan insufficiency in critically ill patients to cirrhotic participants [29, 30].

According to Demirturk et al., variceal rupture is assumed to be caused by H. pylori infection, which causes mucosal injury. Chronic atrophic gastritis can be caused by H. pylori infection. In individuals with persistent H. pylori infection, gastric mucosal atrophy progresses and reduces stomach acid secretion [31]. It causes a local and general increase of proinflammatory cytokines, including interleukins, interferon-β, and tumor necrosis factor-α [18]. In H. pylori-induced follicular gastritis, the mechanisms that cause increased gastrin secretion are unknown. Local alkalization of ammonia generated by H. pylori urease in the region of G cells is thought to increase gastrin release [32]. Another possibility is that H. pylori infection lowers the number of antral D cells and somatostatin levels, resulting in a lack of physiologic somatostatin inhibition on G cells and hence increased gastrin release [33], which act on the production of pepsinogen that has been significantly linked with increased risk of esophagogastric variceal bleeding in this study. In contrast, Quintero et al. found that bleeding in cirrhotic patients due to gastric mucosal vascular ectasias was associated with hypergastrinemia and low serum levels of pepsinogen I. This conflict is attributed to the difference in the studied group, cause of cirrhosis, and severity of disease [34]. Kolster and his team reported that an elevated level of pepsinogen was associated with a significantly (p < 0.001) higher incidence of duodenal ulcer and gastric bleeding [35].

Based on the baseline of the included patients, there was a significant difference between both groups in terms of HCV treatment status. In both patients with and those without cirrhosis prior to therapy, HCV eradication may minimize the incidence of variceal bleeding during long-term follow-up. HCV eradication may lower the chance of developing cirrhosis in individuals without cirrhosis prior to therapy, which lowers the risk of developing varices and variceal bleeding [36]. Improvements in portal hypertension and a decreased risk of variceal bleeding may result from HCV eradication in cirrhotic individuals because it may halt or even reverse the progression of fibrosis [37].

Patients with minor varices who have not yet bled but are at a higher risk for bleeding are candidates for prophylactic therapy with non-selective beta blockers (NSBBs). For patients with medium- to large-sized varices, NSBBs or endoscopic band ligation are the suggested therapeutic techniques for primary prevention of variceal bleeding. On the other hand, sclerotherapy, shunt surgery, and nitrates are all not recommended [38]. Patients who have resistance to NSBBs or who have contraindications to pharmaceutical treatment may benefit from endoscopic band ligation [39].

We acknowledge that our study has some limitations, including the small sample size and the single-center setting, which may hinder the generalizability of our findings. In addition, many important variables such as MELD score, AST Platelet Ratio Index (APRI), FIB-4 index, and Platelet count-Spleen diameter (PC/SD) ratio were not assessed in this study.

In conclusion, our findings showed that patients with esophagogastric variceal bleeding are typically presented with moderate to severe ascites, lower limb edema, non-palpable liver, splenomegaly, dilated portal vein, advanced Child-Pugh score (B and C), positive H. pylori, reduced hemoglobin, RBCs, platelets, and albumin, and elevated ALT, AST, bilirubin, creatinine, INR, and pepsinogen. The independent predictors of esophagogastric variceal bleeding were ascites, positive H. pylori, Child-Pugh score B and C, pepsinogen level higher than 43.5 μg/l, AST (>54.5), bilirubin (>1.45), and hemoglobin (>11.5). Laboratory investigations are more reliable in predicting variceal bleeding and excluding non-variceal bleeding; however, clinical symptoms should not be neglected, especially H. pylori infection, ascites, and Child-Pugh score. Further studies are required to explore more predictors and markers of esophagogastric variceal bleeding and create a prediction score based on these models.
